# Regulation of the Nucleosome Repeat Length *In Vivo* by the DNA Sequence, Protein Concentrations and Long-Range Interactions

**DOI:** 10.1371/journal.pcbi.1003698

**Published:** 2014-07-03

**Authors:** Daria A. Beshnova, Andrey G. Cherstvy, Yevhen Vainshtein, Vladimir B. Teif

**Affiliations:** 1 Deutsches Krebsforschungszentrum (DKFZ) and BioQuant, Heidelberg, Germany; 2 Institute for Physics and Astronomy, University of Potsdam, Potsdam-Golm, Germany; Ottawa University, Canada

## Abstract

The nucleosome repeat length (NRL) is an integral chromatin property important for its biological functions. Recent experiments revealed several conflicting trends of the NRL dependence on the concentrations of histones and other architectural chromatin proteins, both *in vitro* and *in vivo*, but a systematic theoretical description of NRL as a function of DNA sequence and epigenetic determinants is currently lacking. To address this problem, we have performed an integrative biophysical and bioinformatics analysis in species ranging from yeast to frog to mouse where NRL was studied as a function of various parameters. We show that in simple eukaryotes such as yeast, a lower limit for the NRL value exists, determined by internucleosome interactions and remodeler action. For higher eukaryotes, also the upper limit exists since NRL is an increasing but saturating function of the linker histone concentration. Counterintuitively, smaller H1 variants or non-histone architectural proteins can initiate larger effects on the NRL due to entropic reasons. Furthermore, we demonstrate that different regimes of the NRL dependence on histone concentrations exist depending on whether DNA sequence-specific effects dominate over boundary effects or *vice versa*. We consider several classes of genomic regions with apparently different regimes of the NRL variation. As one extreme, our analysis reveals that the period of oscillations of the nucleosome density around bound RNA polymerase coincides with the period of oscillations of positioning sites of the corresponding DNA sequence. At another extreme, we show that although mouse major satellite repeats intrinsically encode well-defined nucleosome preferences, they have no unique nucleosome arrangement and can undergo a switch between two distinct types of nucleosome positioning.

## Author Summary

The DNA molecule of a human or mouse can be up to two meters long, if stretched. However, it is stored inside the small volume of the nucleus in the living cell. DNA compaction is achieved at different hierarchical levels with the help of a number of architectural proteins. The elementary unit of compaction is the nucleosome, where DNA is wrapped around the protein octamer core. Each nucleosome contains about 147 DNA base pairs; the length of DNA between the neighboring nucleosomes varies from nearly zero to several hundred of base pairs. This variability determines the biological function of the underlying DNA, since some parts of the genome are less compact, and thus potentially actively transcribed, while others are more compact, and their transcription is limited. The DNA distances between neighboring nucleosomes depend on the interaction with many chromatin-associated proteins. The average distance between two neighboring nucleosomes change for different genomic locations and even for the same genomic region in different cell states of the same organism. Here we study these effects and provide their quantitative biophysical description using available experimental data in a number of organisms, ranging from yeast to frog to mouse.

## Introduction

The elementary unit of DNA packaging in the eukaryotic cell is the nucleosome, which consists of ∼147 bp of DNA wrapped around the core histone octamer. The nucleosome is commonly associated with the linker histone H1, this complex being referred to as the chromatosome. In addition, a number of other non-histone architectural proteins present in quantities comparable to histones help to shape chromatin [1]. Since the discovery of the nucleosome as the basic unit of chromatin [2], [3], it become apparent that nucleosomes sometimes form ordered arrays, and scientists were trying to understand the principles governing the regularity and fuzziness of nucleosome arrays *in vivo* [4]–[6], the question which is still far from being understood. In practice, the knowledge of nucleosome positions is required to estimate the accessibility of transcription factors (TFs) to their DNA binding sites, quantify 3D chromatin structure, and ultimately understand the epigenetic regulation of gene expression. For this purpose, 1D lattice descriptions with nucleosome positions characterized by a single genomic coordinate became very popular [7]–[25].

An important chromatin property that determines its biological function is the so-called nucleosome repeat length (NRL), defined as the average distance between the centers of neighboring nucleosomes. NRL can be defined either as a genome-average value, or as an average for a smaller subset of genomic regions. Past studies going back to 1970s showed that, in general, NRL is different for different species and even for different cell types of the same organism [1], [26]. In addition, recent publications reported NRL variations for different genomic regions of the same cell type [27], [28]. An important recent work that compared the NRL around yeast transcription start sites (TSSs) *in vivo* and that for the reconstituted chromatin on the same DNA sequences *in vitro*, has showed that ordered nucleosome positioning arises only in the presence of ATP and chromatin remodelers [29]. Furthermore, it was reported that the NRL determined around yeast TSSs is an invariant value universal for a given wild type yeast strain [29], [30], although it can change when one of chromatin remodelers is missing [31]. These findings are difficult to explain within a simplistic theory of a homogenous 1D lattice gas of nucleosomes, which predicts that NRL is a decreasing function of the nucleosome density on the DNA [32]. In addition, large NRL changes have been determined experimentally in higher eukaryotes as a function of histone concentration, including “*in vivo* titration” experiments in *Xenopus oocytes*, where exogenous concentrations of H1 variants were varied in a controlled way [33], as well as experiments in the living mouse cells with knock out of certain H1 variants [34]. In the latter case, a linear dependence of the NRL on the linker-to-core histone ratio was found, which is consistent with electrostatic models attributing NRL changes to the DNA charge neutralization [34]–[36]. There are many other abundant architectural chromatin proteins in addition to H1, such as, e.g. the high-mobility group (HMG)-proteins [37], or DNA methyl-binding proteins, MeCP2, which are expressed at near core-histone levels in neurons and globally alter the chromatin state [38]. An interesting recent work has showed that genomic region-specific NRL differences in the Drosophila genome can be accounted for by the HMGD1 to H1 ratio in those regions. Both HMGD1 and H1 bind DNA electrostatically, but affect NRL in the opposite ways. Thus, specific cooperative interactions between architectural proteins and nucleosomes need to be taken into account on top of nonspecific electrostatic effects. In addition, NRL can change as a function of the 3D structure of the chromatin fiber [39], which indicates that long-range internucleosome interactions also need to be considered.

A biophysical theory quantitatively incorporating the interplay of the above mentioned effects together with the DNA sequence to predict local NRL changes is currently missing. In order to develop such a theory, we depart from the previous 1D lattice models for nucleosome positioning, and focus on the next levels of complexity to study the effects on the NRL of the chromatin fiber structure and contributions of binding of linker histones and non-histone architectural proteins, as well as the binding of sequence-specific TFs. We start with a systematic analysis of the parameter space of the updated lattice model and determine how NRL depends on the short- and long-range interactions between nucleosomes, DNA unwrapping from the nucleosome and the concentration of core and linker histones. In the following section we apply our model to explain the seemingly controversial published dependencies of NRL on the concentration of core and linker histones *in vivo* and *in vitro* [29], [33], [34]. Finally, we combine biophysical and bioinformatics analysis with our high-resolution nucleosome positioning data in mouse embryonic stem cells to show that while direct and indirect sequence-specific effects dominate at *cis*-regulatory regions, the NRL of the constitutive heterochromatin appears to be mostly determined by the chromatin context rather than the repetitive DNA sequence.

## Results and Discussion

### Model

Classical 1D lattice models for DNA-ligand binding go back to 1970s (for review see [15], [40]). Together with a related class of lattice models for the DNA helix-coil transition, these are the descendants of the famous Ising model of ferromagnetism [41]. A common theme of these models is that the DNA is considered as a lattice of units (base pairs, bps), which can be in different states (e.g. bound or not bound by a given protein type), and the state of a given lattice unit can affect the states of its neighbors. An important conclusion from these studies is that non-specific binding of a large ligand to DNA in the presence of boundaries (close to the ends of the DNA segment or close to some other obstacles) results in a non-random periodic oscillation of the ligand binding probability [42]. In 1988, Kornberg and Stryer proposed that a similar effect accounts for regular oscillations of preferred nucleosome positions in the genome [43]. The model of Kornberg and Stryer assumed a “1D gas” of self-excluding histone octamers which can freely diffuse along the DNA [43]. This model had only one input parameter, the nucleosome density. Later, it was generalized in the spirit of classical ligand-DNA binding models [44] to include basic thermodynamic parameters such as the histone octamer binding constant, the length of the nucleoprotein particle in units of DNA base pairs (bp), and the contact cooperativity parameter for the interaction between nucleosomes [45]. Following bioinformatic studies based on high-throughput sequencing provided compelling arguments to introduce a discrete distribution of sizes of the linker DNA between nucleosomes [17] and long-range nucleosome-nucleosome interactions [18], [46]. Additionally, arguments have been put forward that the model should be modified to take into account that the nucleosome core particle is not a static structure and some plasticity and partial DNA unwrapping from the histone octamer core can allow and facilitate the binding of TFs to the nucleosome-associated DNA [47]–[50]. Correspondingly, generic lattice models were adapted to include nucleosome unwrapping [16], and DNA unwrapping, which was shown to be an essential feature required for an adequate analysis of the available experimental data on nucleosome positioning [7], [10], [16]. We will start from the model that includes all the features mentioned above. On top of it, we will take into account nucleosome interactions with each other and with chromatin architectural proteins (H1, HMG1, etc).

**Figure 1:**
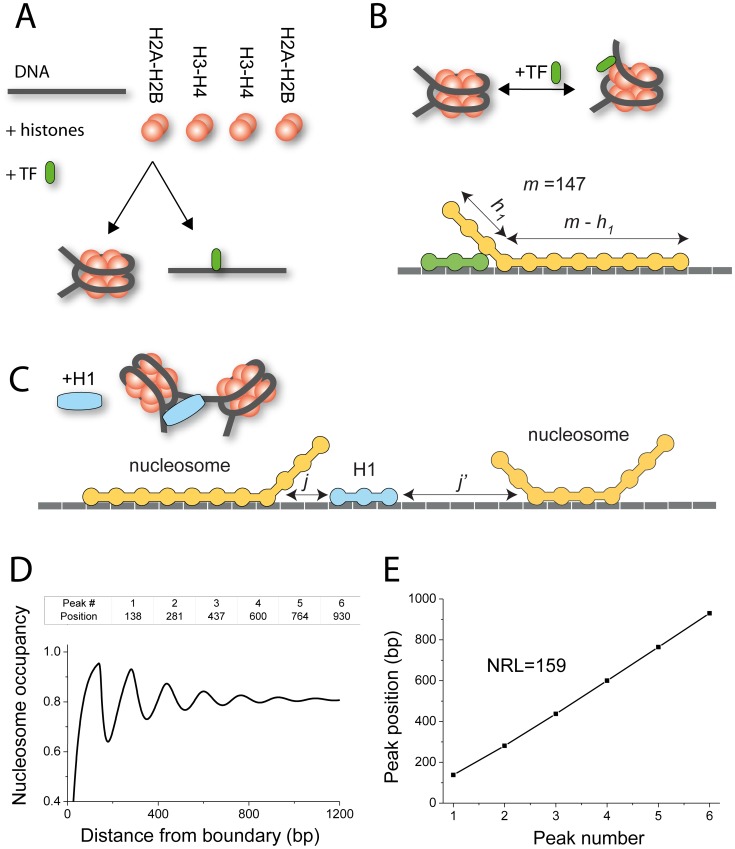
Lattice models to calculate nucleosome/TF binding landscapes in chromatin. A) All-or-none models require that a DNA region is either within a nucleosome or bound by a transcription factor. B) Advanced view on co-binding of a TF and histone octamer to the same DNA region (top), and the corresponding lattice model (bottom), which takes into account the possibility of partial nucleosome unwrapping. C) Taking into account linker histones requires the introduction of long-range interactions between DNA-bound proteins. D), E) The scheme of the method of NRL calculation. Firstly, the oscillations of the nucleosome density are plotted around the boundary of interest (for example, an end of the DNA segment would be appropriate as a boundary). Then the coordinates of the peaks from (D) are collected and fitted with a linear function. The slope of the line in (E) determines the NRL.

The scheme of our generalized 1D lattice model for nucleosome arrangement is presented in Figure 1. In the framework of this model, genomic DNA is represented as a 1D lattice of units numbered by index *n*, each of which can be either bound by any of *f* protein species or remain unoccupied. Each protein type *g* is characterized by its size *m*(*g*) in terms of the number of DNA base pairs covered upon its binding, the concentration of free protein, *c*_0_(*g*), and DNA sequence-specific binding constant, *K*(*n*, *g*). In principle, the histone octamer is treated as just one of the many possible types of DNA-binding protein complexes. The use of the binding constant for the histone octamer does not mean that the complex freely binds and unbinds at equilibrium. Indeed, it is known that the free thermal sliding of the histone octamer along the DNA is limited at physiological conditions [51], and most nucleosome repositioning events happen *in vivo* actively with the help of ATP-dependent remodelers and histone chaperones, which decrease energy barriers for nucleosome translocations and effectively help to equilibrate the system. Remodelers can have their context- and cell type-dependent rules [20], [52], but since these rules are not well known in practice, we can assume that these rules are already taken into account in the experimentally measured cell type-specific nucleosome distributions. Thus, the “equilibration” to get such a nucleosome distribution is actually a non-equilibrium process. Still, one can treat it as a collective equilibrium in an ensemble of many identical cells or for many instances of the cell at different time points [53].

The nucleosome core particle (NCP) is characterized by a nominal size *m*(NCP) = 147 bp, but the model allows its unwrapping by *h*_1_ and *h*_2_ bp from each end, up to a total allowed unwrapping length of *h*_max_≥*h*_1_+*h*_2_. The linker histone H1 (or any other architectural protein) is allowed to bind both the free DNA and the nucleosome. The model assumes, for mathematical simplicity, that the binding takes place at the DNA lattice units free from other proteins. The physical connectivity between the linker histone and nucleosome (and in general for other protein binders) is accounted for by the interaction potential *w*(*L*,*g*_1_,*g*_2_), where *L* is the distance along the DNA between proteins *g*_1_ and *g*_2_. In the limiting case in the absence of protein-protein separation (*L* = 0), *w*(0,*g*_1_,*g*_2_) has a meaning of a standard McGhee-von Hippel contact cooperativity [44], [54]. Another limiting case of *w*(*L*<*V*,*g*_1_,*g*_2_) = 0 corresponds to the long-range anti-cooperativity [55] (for a given protein pair, protein binding is prohibited within *L*<*V* bp from another bound protein). In particular, since H1 interacts mostly with the nucleosome [56], this has to be reflected by a high value of the contact cooperativity parameter *w*(0,H1,NCP). An intermediate case *w* = *f*(*L*) has been considered elsewhere [57].

The lattice model illustrated in Figure 1 can be solved mathematically either using dynamic programming or the transfer matrix formalism [14], [16], [40], [53]. Here we have performed the calculations of nucleosome binding maps using our software suite *TFnuc* [53], which is based on the dynamic programming algorithm developed in our previous publication [40]. See Supplementary Materials for the details of the computational implementation. *TFnuc* takes as input concentrations of DNA and DNA-binding proteins and position weight matrices (PWMs) for all the TFs studied, as well as the thermodynamic parameters listed above, which define the properties of the interaction model. As the output, TFnuc calculates binding probabilities *c*(*g, n*) for each protein type *g* at a genomic position *n* taking into account the presence of all other proteins and nucleosomes. The NRL for a given genomic region can be then determined, following our previous work [28], from a linear fit of the nucleosome occupancy peak positions versus the corresponding peak numbers (Figure 1D and E).

### Non-sequence-specific effects of thermodynamic parameters on the NRL

**Figure 2:**
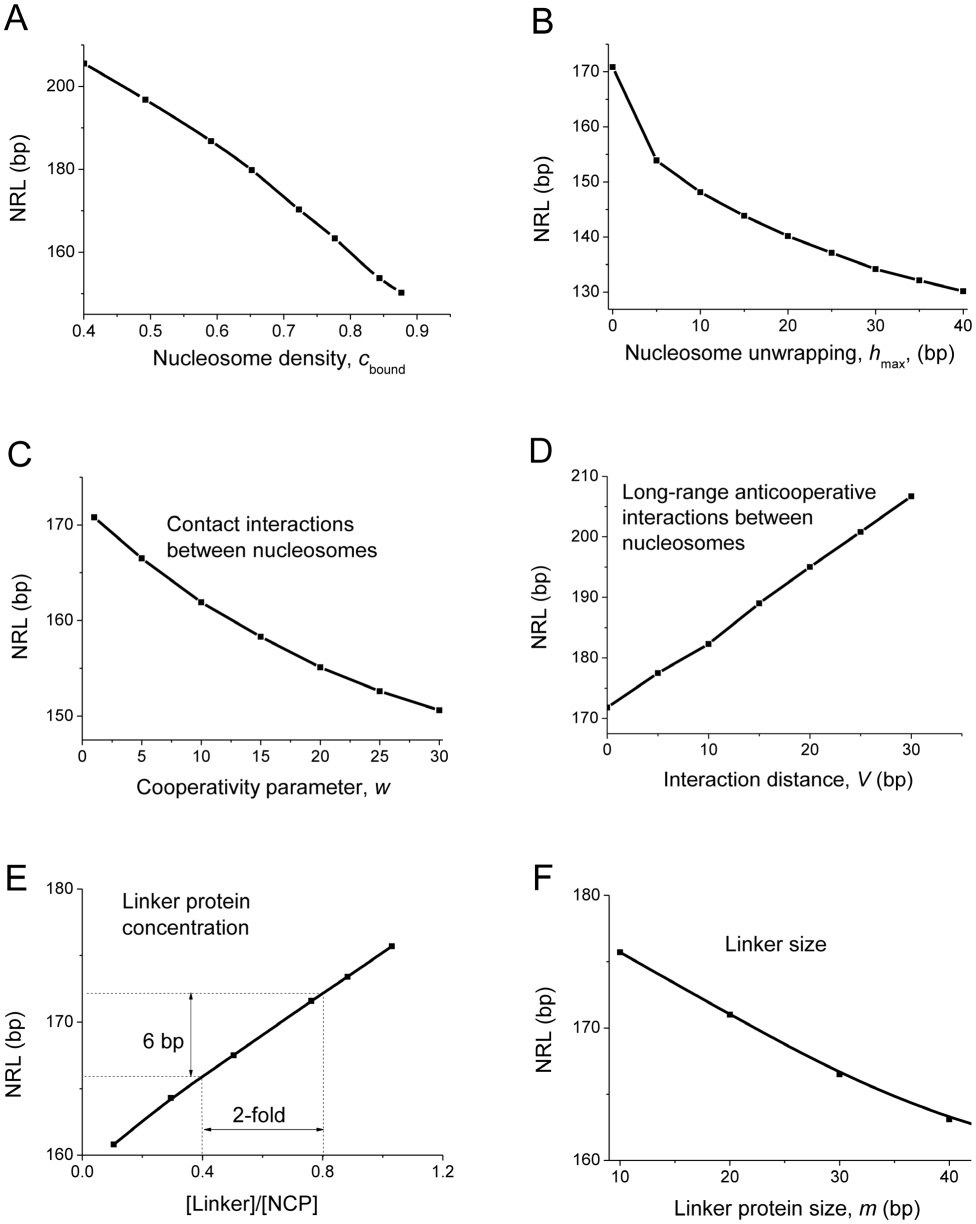
NRL as a function of thermodynamic parameters such as the concentration of bound nucleosome core particles (A), maximum allowed length of DNA unwrapping from the nucleosome (B), contact cooperativity between nucleosomes (C), long-range anti-cooperativity between neighboring nucleosomes (D), ratio of linker histone per nucleosome (with *m*(linker) = 15 bp) (E), and the effective size of the linker protein in terms of covered DNA base pairs (F). Unless stated otherwise in the figure, the following parameters were used: *K*_NCP_* *c*_0_(NCP) = 0.7; *K*_linker_ = 2·10^9^ M^−1^.

Figure 2 shows the results of calculations of the NRL as a function of different thermodynamic parameters, assuming that there are no sequence-specific preferences of histone octamer binding to the DNA. We make a number of general conclusions based on these calculations:

#### 1) Higher nucleosome density leads to shorter NRLs

Figure 2A demonstrates that as the nucleosome density increases, NRL sharply decreases down to the minimum size defined by neighboring nucleosome-nucleosome interactions (see below). It is this decrease of NRL with the nucleosome density predicted by the theory but not observed in the yeast chromatin reconstitution experiments [29], that has lead to the questioning of the validity of the Kornberg-Stryer statistical model for this system [58]. Therefore, it is important to note that the assumption of non-sequence-specific binding is not valid for the case when DNA sequence-specificity dominates (e.g. near TSSs), while it is a reasonable approximation when the genome-wide NRL is calculated, in which case all sequence-specific effects are averaged out. Interestingly, the magnitude of the effect predicted in Figure 2A (∼10 bp NRL decrease corresponding to a ∼10% nucleosome density increase) is consistent with recent remodeler knockout experiments in *S. pombe* [31].

#### 2) Partial nucleosome unwrapping shortens NRL

Figure 2B shows that when nucleosome unwrapping is allowed, NRL decreases nonlinearly as a function of the maximum allowed unwrapping length *h*_max_. The non-linearity arises due to the fact that not all nucleosomes adopt the state with the largest possible unwrapping; some nucleosomes in the ensemble stay completely intact, while some others have just a few base pairs unwrapped (due to the thermal distribution of NCP unwrapping lengths). More unwrapping requires more energy to break attractive histone-DNA contacts, which is only partially compensated by the favorable entropy increase [16]. Previously, nucleosome unwrapping was shown to be essential to describe *in vitro* AFM data [16], *in vivo* genome-wide nucleosome distribution in yeast [7], and the effect of nucleosomes on the activating enhancer function in drosophila [14]. Thus, nucleosome unwrapping is an essential feature of our current model and its effect on the NRL found in Figure 2B needs to be taken into account.

#### 3) NRL decreases due to the contact cooperativity and increases due to the long-range anticooperativity between neighboring nucleosomes

Figure 2C shows that the effect of the contact cooperativity between neighboring nucleosomes leads to the NRL decrease with the increase of the contact cooperativity parameter *w*(*L* = 0). On the other hand, introducing long-range anticooperative interactions leads to a linear increase of the NRL as a function of the length of prohibitive interactions *V* (Figure 2D). The long-range interaction potential *w*(*L*) can be introduced in any form in the framework of this model. The calculations in Figure 2D tested the limiting case of long-range anticooperativity when nucleosome distances shorter than *V* are prohibited (*w*(*L*<*V*) = 0). Intermediate situations with length-dependent interaction potential would lead to a more complicated behavior, which can be also studied with the help of this model.

#### 4) Non-core-histone protein intercalation between nucleosomes increases NRL

Now let us consider linker histones H1 (or their variants such as H5, or other chromatin proteins such as HMGN1), as schematically depicted in Figure 1C. In the 1D lattice model, the linker histone is assumed not only to bind the nucleosome, but also to cover several free DNA lattice units between nucleosomes, depending on the size of this protein. A proper affinity of the linker protein (“linker”) to the nucleosome core particle (“NCP”) is introduced by the interaction potential *w*(*L*, linker, NCP). Effectively, linker proteins introduce additional nucleosome-nucleosome interactions (e.g. repulsive steric interactions and attractive electrostatic interactions). The results of our calculations shown in Figure 2E suggest that the effect of linker proteins is quite different from the effect of direct nucleosome-nucleosome interactions considered in panels 2C and 2D. The major difference is that the effect of linker proteins is concentration-dependent. Figure 2E shows that when the linker protein concentration is large, it has a significant effect of the NRL. In particular, a two-fold change of the linker-to-core histone ratio of molar concentrations leads to an experimentally detectable 6 bp NRL change.

#### 5) Smaller linker proteins can introduce stronger effects on the NRL

Figure 2F shows that the size of the linker histone (or other non-histone players nonspecifically binding the nucleosome and the DNA linker between nucleosomes) is quite important for its ability to alter the NRL. Counterintuitively, smaller proteins appear to be more effective in increasing the NRL due to larger configurational entropy of rearrangements of bound proteins along the DNA. The latter effect was obtained assuming that H1-DNA binding affinity does not depend on the H1 size, which is not necessarily the case if the electrostatics of DNA-histone binding prevails [35]. The finding that smaller proteins can exert larger steric effect on the NRL is in line with *in vitro* DNA condensation experiments, which have established that although cationic ligands with higher charge are better DNA condensing agents, smaller cations have stronger DNA condensing propensity when ligands of the same charge are considered, such as e.g. linear flexible polyamines and multivalent metal ions [59]. In a similar way, Blank and Becker reported that the effect of multivalent binders including metal ions, polyamines and H1 on the NRL increases with their charge [36]. One should expect that proteins or polyamines with the same charge have different properties depending on their size according to Figure 2F. Since this effect is directly testable, it would be interesting to confirm it experimentally.

### Effects of nucleosome unwrapping and long-range interactions at yeast promoters

**Figure 3:**
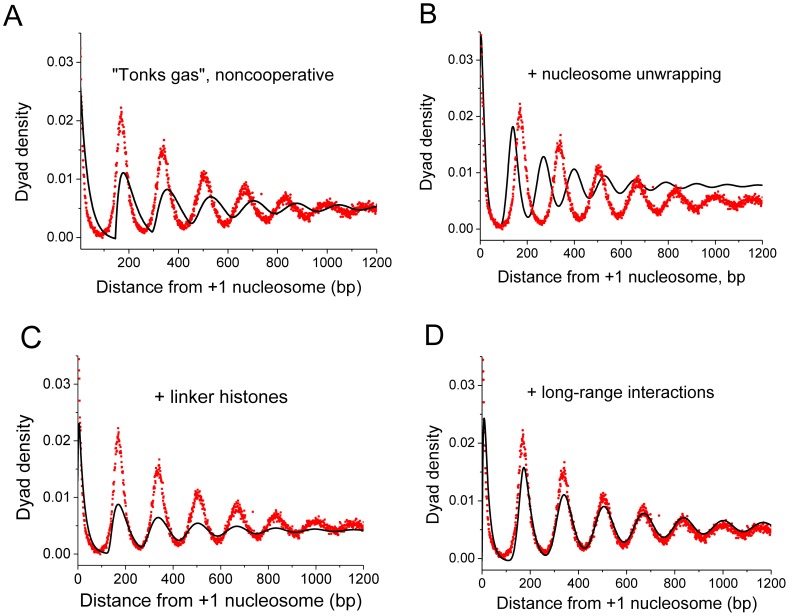
Nucleosome occupancy patterns around TSS in *S. cerevisiae* explained by the lattice binding model. Dots correspond to the experimental data [29]; straight lines are the nucleosome density patterns estimated by *TFnuc* algorithm with the following model parameters: A) “Tonks gas” model: *N* = 4000 bp, *K*_NCP_ = 3·10^6^ M^−1^; *c*_0_(NCP) = 10^−6^ M; B) Nucleosome unwrapping model: *K*_NCP_ = 3·10^6^ M^−1^; *c*_0_(NCP) = 10^−6^ M; *h*_max_ = 40; C) Linker histone model: *K*_NCP_ = 1·10^5^ M^−1^; *c*_0_(NCP) = 10^−6^ M; *K*_linker_ = 9·10^5^ M^−1^; *c*_0_(linker) = 10^−6^ M; *m*(linker) = 15 bp; *h*_max_ = 15 bp; D) Long-range interaction model: *K*_NCP_ = 3·10^6^ M^−1^; *c*_0_(NCP) = 10^−6^ M; *h*_max_ = 40 bp; *w*(0:30, NCP, NCP) = 0.

We will start our analysis of sequence-specific NRL effects from the description of the nucleosome arrangement around *Saccharomyces cerevisiae* TSSs [29], the system which has already become a standard benchmark for this type of models [7], [10], [32], [58], [60], [61]. Figure 3A shows the average experimental nucleosome distribution and the corresponding prediction from the Kornberg-Stryer model where nucleosomes are modeled by stiff rod-like particles non-cooperatively binding the DNA and protecting 147 bp from other nucleosomes and proteins (Figure 1A). While revealing the expected oscillatory occupancy pattern, this model fails to describe the experimental data quantitatively. In particular, the first dip of the nucleosome density at position around 147 bp is too sharp in comparison with the experimental curve. This discrepancy has been noted by Riposo and Mozziconacci [60], which they tried to solve mathematically via blurring the precise nucleosome dyad positions by a finite-width Gaussian distribution. A more physically motivated tuning of the model was used to correct for this effect in a recent study by Mobius et al. [10]. In the latter work it was shown that allowing DNA unwrapping from the nucleosome makes the distribution smoother and more resembling the experimental one. Yet, the calculation in Figure 3B shows that extending the model to allow nucleosome unwrapping brings another problem. Nucleosome unwrapping makes the nucleosome effectively “shorter”, which in turn shortens the NRL. In the frame of our model we have the option to fine-tune the parameter landscape by changing the histone octamer affinity to the DNA, the concentrations of core and linker and the contact nucleosome-nucleosome cooperativity parameter. However, none of these new elements of the model allowed fitting the experimental curve adequately. In particular, Figure 3C shows that the introduction of the linker histone H1, while allowing properly changing the NRL, does not lead to the proper shapes of the peaks of the nucleosome density. This is not surprising, since yeast does not have H1 [62], and the related protein Hho1 is probably not involved in determining NRL [63], [64].

The only model change that helped us arriving to the experimentally observed yeast TSS nucleosome distribution was the introduction of the long-range anticooperative interactions between nucleosomes, as schematically shown in Figure 1C. Figure 3D shows that the model with *w*(*L*<30 bp) = 0 and *h*_max_ = 40 bp allows describing the nucleosome distribution around yeast TSSs quite well. Note that the experimentally determined average distance between nucleosomes is around 15 bp, which is less than *V* = 30 bp due to partial nucleosome unwrapping. Several previous publications have also encountered the problem that the theoretically predicted NRL is too short for this experimental system, and attempted to solve it by empirically assuming that nucleosomes cannot form closer than a certain distance (e.g. 10 DNA bp) from the end of one nucleosome to the beginning of the consecutive one [17], [18], or even assuming that the nucleosome core particle contains 158 bp instead of the commonly perceived 147 bp [61]. However, the latter model fails to describe another yeast strain, *Schizosaccharomyces pombe*, which has NRL = 154 bp, leaving only 7 bp between neighboring nucleosomes [65]. The 3D structure of the nucleosome is essentially the same in these species, so if one postulates a nucleosome consisting of 158 bp in *S. cerevisiae*, then why *S. pombe* would have different nucleosomes? Therefore, instead of setting the fixed-geometry contact interaction between nucleosome core particles, our model only assumes that nucleosomes need to overcome a certain energy barrier to position themselves closer than a certain distance at a given genomic region. Thus the lower NRL limit determined by long-range interactions is different for different cell types depending on the thermodynamic conditions.

What can be the source of such long-range interactions? One of the differences between *S. pombe* and *S. cerevisiae* is that *S. pombe* lacks ISWI remodelers, which require at least a 10 bp DNA linker for their nucleosome-spacing activity, but has an expanded CHD remodeler family instead [66]. Different remodeler composition could explain NRL differences in different species; in addition, the co-evolution of the remodeling system and the nucleosomal DNA code might determine that the DNA sequence is also adapted to this or that NRL in different regions in different species. Another possible justification for long-range nucleosome-nucleosome interactions could be a specific structural nucleosome arrangement in the chromatin fiber [67]. For instance, the geometries and nucleosome axial densities of the *in vitro* reconstituted chromatin fibers in the presence of linker H5 histones were shown to be dramatically different for the NRL of 167 and 197 bp [39]. Longer DNA linkers enable easier compaction of the chromatin [68], while for short linkers, on the contrary, the elastic penalty of the linker DNA bending can become too large [69].

### *In vivo* NRL is an increasing but saturating function of H1 concentration

**Figure 4:**
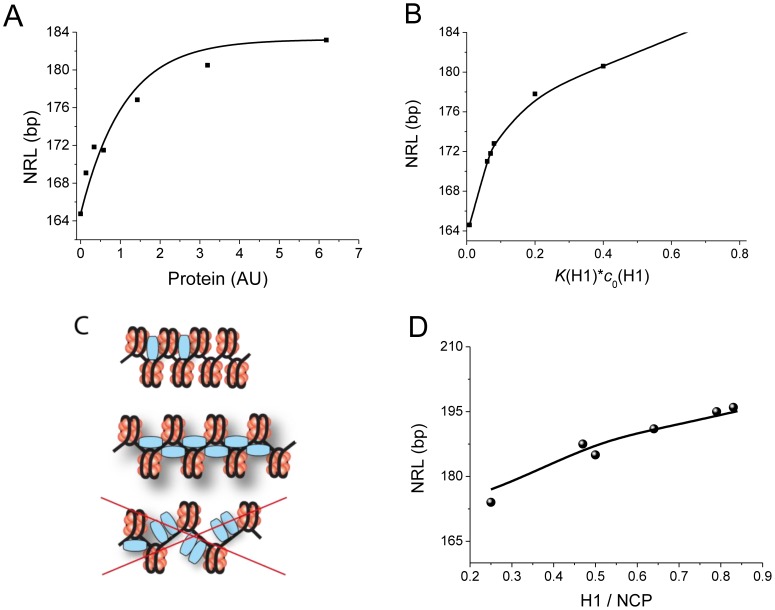
NRL dependence on the concentration of linker histones. A) Experimentally determined NRL as a function of the concentration of histone hH1.4 [33]. B) Theoretically predicted NRL as a function of H1 activity. *K*_H1_ – binding constant; [H1] – concentration of free H1 in solution; *m*(H1) = 15 bp. C) A scheme illustrating the refined model for nucleosome-H1 arrangement: different configurations of bound H1 around nucleosome are allowed, but not more than a critical number of H1 per nucleosome. D) Dots - experimental NRL data from Woodcock et al. [34] for different mouse cell types. Solid line - theoretical prediction. *m*(H1) = 15 bp, *K*_H1_* *c*_0_(H1) = 0.0035, *w*(0, NCP, NCP) = 11.

Let us compare predictions of the model with available experimental dependences of NRL on the concentration of linker histone H1, [H1]. We will use two experimental examples. In the first example, Oberg and coauthors have systematically studied the effect of the concentration of different histone variants on the NRL [33]. In their system, linker histone concentration was “titrated” exogenously in living cells, *Xenopus oocytes*. They have found that for all the histone variants an increase of the NRL with increasing H1 concentration was observed, which saturated at a certain value (Figure 4A). The fact that NRL is a smooth function of [H1] speaks against a purely DNA-sequence or remodeler-determined NRL formation mechanism in this case. In addition, a simple competitive model where H1 binds the nucleosomal DNA and excludes nucleosomes cannot explain the saturating behavior of NRL as a function of [H1]. To recapitulate this feature, we have introduced in the model a limiting case of cooperative binding: we prohibit H1 binding if there is no nucleosome in its vicinity. This model keeps nucleosome-H1 and nucleosome-H1-nucleosome distances flexible, but allows not more than one H1 per nucleosome to be bound (Figure 1C). Figure 4B shows that this model leads to the NRL saturation at high H1 concentrations. Furthermore, this modified model predicts the correct slope of the curve and the correct saturation level in comparison with the experiment of Oberg et al. [33]. This leads us to the refined lattice model for H1-nucleosome interaction, where nucleosomes can bind a small number of H1 molecules, or accommodate different types of nucleosome-H1-nucleosome connectivity, but only less than a critical number of H1 molecules per nucleosome is allowed (one H1 per nucleosome in Figure 4C). This model is consistent with recent simulations [70] and structural data [62] which suggest that H1 binds to distinct sites almost exclusively in the nucleosome entry/exit area.

In the second experimental example, Woodcock and colleagues investigated the effect of H1/NCP ratio in different mouse cells where some of H1 variants were knocked out [34]. The authors have found a mostly linear dependence of the NRL on the [H1]/[NCP] ratio, which they have interpreted in terms of the electrostatic screening and mutual charge neutralization of the negative DNA charge by basic histones H1. A similar behavior was also found by Blank and Becker for various smaller charged molecules such as metal cations and polyamines [36]. Electrostatics is also believed to play a role in the regulation of chromatin states through histone modifications [71]. Here, our calculations performed in the frame of the lattice model allow to recapitulate the experimentally found NRL dependence on the H1/NCP ratio (Figure 4D). Interestingly, the linear regime observed by Woodcock and colleagues represents only a fraction of the interval of H1 concentrations, where the overall saturating dependence of NRL on the H1 concentration was found by Oberg et al. [33] (Figure 4A, B). Thus, our model is consistent both with the Oberg et al. saturation behavior and with the Woodcock at al. and the Blank and Becker's concept of the electrostatic screening, and provides a deeper understanding and NRL predictions for the wide interval of histone concentrations. This model is not limited to the *in vitro* system consisting of just the DNA and histones. Importantly, it is also readily applicable to more complicated systems where histones are complemented by non-histone chromatin proteins such as HMGN1. A recent study where NRL differences in different genome regions were correlated with the local HMGN1/H1 composition provides an example of this kind [37].

### Sequence-dependent nucleosome positioning at mammalian *cis*-regulatory regions

Let us now return to the sequence-specific NRL effects at genomic regions. Korber and colleagues have noted that the statistical model of Kornberg and Stryer [43] predicts a pronounced NRL change with the increase of the nucleosome density (as in Figure 2A), which they did not observe experimentally upon reconstituting nucleosomes at the DNA sequences enclosing yeast TSSs [29], [58]. Furthermore, subsequent experimental work of Celona et al. [30] showed that only weak nucleosomes are being removed upon core histone depletion, while strong nucleosomes remain at their places, effectively keeping the NRL independent of the core histone concentration. In line with this, the authors of a recent Monte Carlo simulation hypothesize that DNA sequence-specific effects have the primary role on nucleosome positioning near TSSs [72]. To check this hypothesis in a more general context, we have utilized the nucleosome positioning data measured by MNase-seq for mouse embryonic stem cells (mESCs) [28].

**Figure 5:**
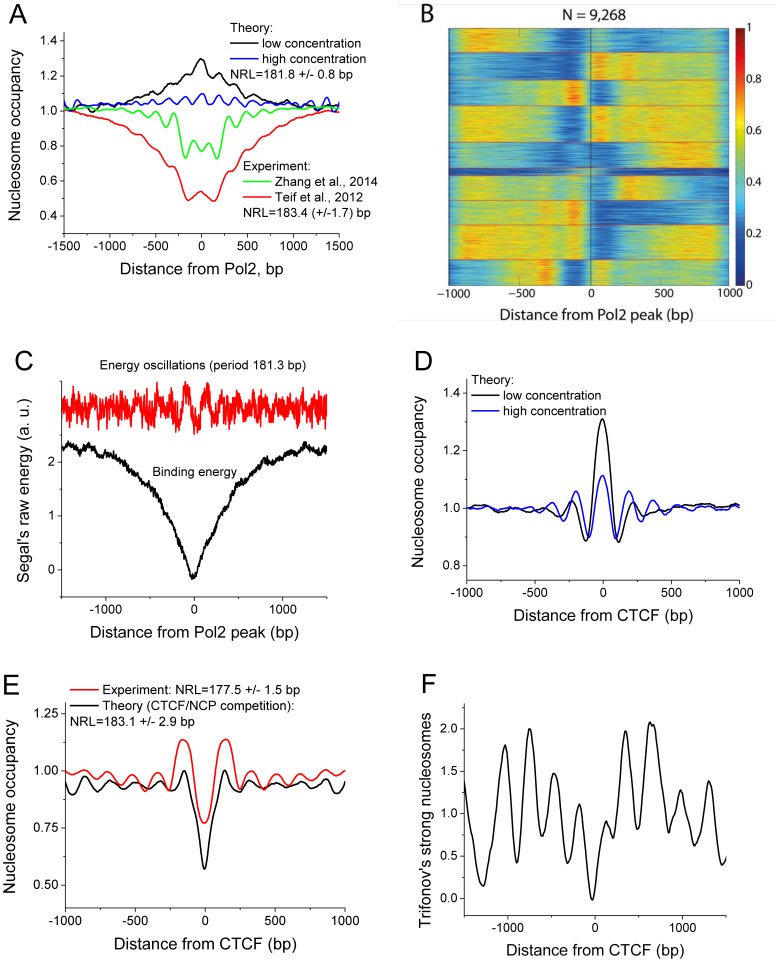
Nucleosome oscillations around CTCF and Pol2 ChIP-seq peaks reveal DNA sequence modulation. A) Red and green lines - experimental nucleosomes in ESCs around Pol2 sites using MNase-seq data from [28] and [73], correspondingly. Black and blue lines - average nucleosome occupancies around Pol2 ChIP-seq peaks predicted from the DNA sequence without competition with Pol2, at different core histone concentrations: *K*_NCP_*c_0_(NCP) = 1.4·10^−6^ and *K*_NCP_*c_0_(NCP) = 1.5 respectively. B) Heat map of the nucleosome density [73] for all individual genomic regions used in the calculation of the average profile in panel A. C) Raw energy of nucleosome formation averaged for the same genomic regions as in A using the method of Kaplan et al [13] (black line), and the difference between the raw energy and its fit with the 90^th^ power polynomial regression, followed by the Fourier transformation. Changing the power of the polynomial regression in the range >50 did not affect the calculated NRL. D) Theoretical nucleosome occupancies in ESCs around CTCF sites predicted from sequence as in (A), black line: *K*_NCP_*c_0_(NCP) = 1.4·10^−6^, blue line: *K*_NCP_*c_0_(NCP) = 1.5. E) Red line - experimental nucleosome occupancies in ESCs around CTCF sites. Black line - theoretical nucleosome occupancies predicted from DNA sequence including competition with CTCF. *h*_max_ = 40 bp; *V* = 40 bp *K*_NCP_*c_0_(NCP) = 0.2; *K*_CTCF_*c_0_(CTCF) = 0.5. F) The probability to find a strong Trifonov's nucleosome determined by the (R_5_Y_5_)_11_ pattern, as a function of the distance from CTCF.

Figure 5A shows that the average nucleosome landscape in mESCs calculated for two different experimental nucleosome datasets from Refs. [28] and [73] around bound RNA polymerase (Pol2) from Ref. [74] is characterized by a strong depletion of the nucleosome density in the region (−1000 bp; +1000 bp) around Pol2 peaks. This is not surprising since most stalled Pol2 reside near the TSS, which is known to be nucleosome-depleted [28]. The ∼1 kb nucleosome depletion is combined with the smaller oscillations of the nucleosome coverage centered at the bound Pol2 peaks (the red and green lines in panel 5A). Panel 5B shows the heat map of the nucleosome density for each of the individual genomic regions used in the calculation of the average profile in panel 5B. This heat map also reveals oscillations of the nucleosome density. To check whether these oscillations are only determined by the TSS-induced boundary or also reflected by the DNA sequence, we have calculated nucleosome distributions using the DNA sequence preferences of histone octamer predicted by Segal and coauthors [13] (black and blue lines in panel 5A). Surprisingly, this nucleosome pattern also exhibits pronounced oscillations. Furthermore, the NRL hard-wired in the DNA sequence (181.8 bp) appears to be quite similar to the NRL found experimentally for these regions (183.4 bp). While the oscillatory pattern was similar, the ∼1 kb depletion was not recovered in the calculations; instead, the theoretical nucleosome landscape calculated for a very small core histone concentration shows average nucleosome enrichment rather than depletion around bound Pol2 sites. When core histone concentration was increased, we obtained even more pronounced oscillations at an overall a flatter landscape. Panel 5C shows the average raw energy of nucleosome formation predicted by the algorithm of Segal and coauthors around Pol2 bound genomic regions [13]. The raw energy has a ∼1 kb dip (the nucleosome affinity is the inverse value, which has a peak, explaining the predicted nucleosome enrichment in Figure 5A). The oscillations of the energy are not visually apparent from the black curve in Figure 5C. To make the oscillations more pronounced, we have calculated the difference between the average raw energy and its fit with the polynomial regression. As a result we obtained the signal with visually detectable oscillations (red line in Figure 5C). This was further Fourier-transformed, which revealed the period of oscillations, 181.3 bp, consistent with panel 5A. These DNA sequence oscillations explain the unexpected result of the nucleosome periodicity predicted from the sequence in Figure 5A. Importantly, the magnitude of the energy oscillations in Figure 5C is very small for each individual base pair. Yet, these lead to the appreciable oscillations of the nucleosome density in Figure 5A due to synergistic effects: firstly, for each individual nucleosome the contributions of each of the 147 base pairs are added up (if in phase), and secondly, neighboring nucleosomes affect each other through excluded-volume interactions, resulting in the nonlocal effects of local DNA sequence variations. On the other hand, it is not possible to exclude the possibility that these oscillations of the DNA sequence appeared as a consequence rather than the cause of formation of ordered nucleosome arrays through the co-evolution of nucleosome arrangement and the underlying DNA sequence. Interestingly, the energy of nucleosome formation predicted by another algorithm, nuScore [75], shows the same ∼1 kb dip, but no detectable oscillations (Figure S1).

As another test case, we have considered NRL around CTCF binding sites in mESCs. CTCF is a zinc-finger protein, which binds to its ∼20 bp recognition motif in ∼40,000 sites throughout the mouse genome. CTCF is implicated in the insulator functions (isolating expressed versus non expressed regions, etc), and is known to be involved in the formation of chromatin loops maintaining 3D chromatin structure [76]. Figure 5D shows our calculation of the average nucleosome landscape around bound CTCF sites in mESCs without taking into account the competition of CTCF with nucleosomes. As noted previously [27], the nucleosome affinity predicted from the DNA sequence at CTCF binding sites is higher than the average. Recently it was shown that differential CTCF binding during stem cell development is determined by the competition with nucleosomes [77]. In line with this, many studies assumed that nucleosome oscillations around CTCF are solely due to their statistical positioning by the boundary created by CTCF. Here, our calculation in Figure 5D shows that the oscillations of nucleosome density can be predicted also from the DNA sequence around validated CTCF binding sites in mESCs without taking into account CTCF binding *per se*. The latter calculation assumes that there is no competition of nucleosome with CTCF. When the binding competition of CTCT with nucleosomes is taken into account, we predict nucleosome depletion instead of enrichment and still measurable oscillations of the nucleosome density (Figure 5E, black line), in agreement with our experimentally observed nucleosome landscapes (Figure 5E, red line). Interestingly, the NRL around CTCF sites observed in the experiment (177.5 bp in Panel 5E, red line) is similar to the NRL predicted without nucleosome/CTCF competition (176.5 in Panel 5D), rather than the NRL that is predicted with CTCF/nucleosome competition (183.1 bp in Panel 5E, black line).

The nucleosome formation energy profiles around CTCF calculated using the methods of Segal and coauthors [13] and Tolstorukov and coauthors [75] did not reveal apparent oscillations, and the Fourier transformation procedure similar to panel 5C did not reveal the energy signal periodicity around CTCF (data not shown). However, sequence-specific features do exist in the vicinity of CTCF sites. One of such features is the presence of “strong nucleosomes” (SNs). SNs is a new class of nucleosomes reflected by a specific symmetry of the underlying DNA sequence, the 10.4 base periodical (RRRRRYYYYY)_11_ pattern, recently discovered by Trifonov and colleagues [78]. About 291 SNs (24% of all SNs predicted in the annotated mouse genome) reside within 10 kb from bound CTCFs [79]. Panel 5F shows the probability to find SNs as a function of the distance from CTCF. The oscillations are very symmetric on average, although in most cases there is exactly one SN in the vicinity of one CTCF (See Supplementary Figure S3). Unlike usual nucleosomes predicted in Figure 4D, strong nucleosomes are not found at the center of CTCF site. Thus, SN provides a second boundary (in addition to CTCF) for positioning the rest of nucleosomes in this region. This situation is different from what would be in the case of a sole CTCF boundary, which explains why the NRL predicted taking into account CTCF/nucleosome competition without the knowledge of SN positions does not coincide with the experimental NRL.

Figure 5 demonstrates that sequence-specific effect play an important role in nucleosome positioning at *cis*-regulatory regions. This can explain the puzzle of the NRL independence of the nucleosome density in the chromatin reconstitution at yeast TSSs [29], [58]. In this regime, DNA sequence preferences dominate over non-specific statistical positioning. The latter effect is further illustrated in Supplementary Figure S4. We have selected an exemplary mouse genomic region around one of the CTCF binding sites. For this region, we have performed calculations of the nucleosome binding maps at different nucleosome densities as indicated in the figure. Due to the dominance of sequence-specific effects, the nucleosome map almost does not change during these calculations in terms of the change in the NRL. Thus, in the DNA-sequence-dependent scenario relevant for the situation at *cis*-regulatory regions, the dependence of the NRL on histone concentration might be not so pronounced.

### Sequence-specificity is not the main NRL determinant in heterochromatin

**Figure 6:**
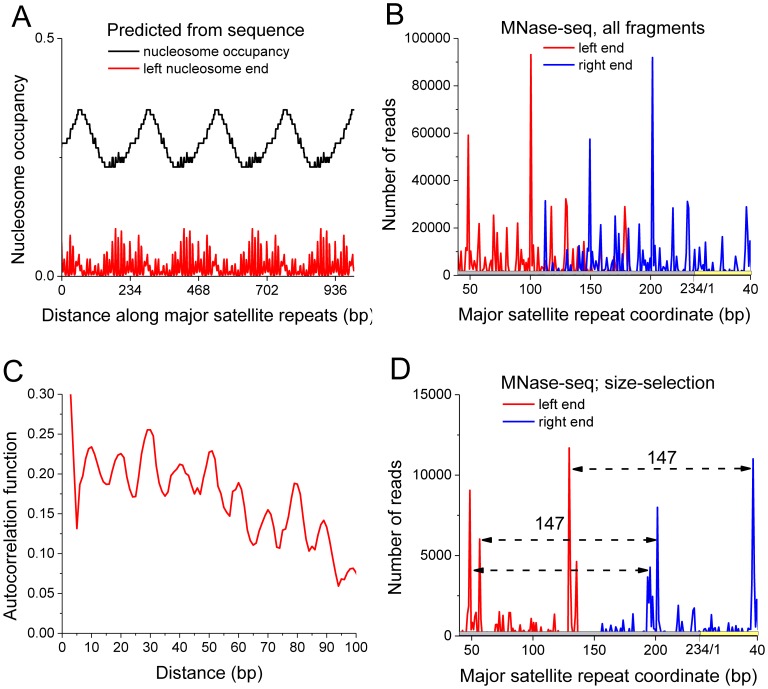
The NRL in mouse pericentric heterochromatin is not determined by the sequence of the major satellite repeats. A) Regular nucleosome positioning around tandems of repeating 234-bp major satellite repeats predicted from the DNA sequence. B) Frequency of the left and right ends of nucleosomal DNA fragments obtained with paired-end MNase-seq [28]. C) Autocorrelation of the nucleosome start site positions from Panel B reveals a 10-bp periodicity. D) Frequency of the left and right ends of MNase-seq nucleosomal DNA fragments size-selected in the interval [145 bp; 150 bp].

A situation opposite to the cis-regulatory regions studied above is encountered in highly compacted, mostly transcriptionally inactive, constitutive heterochromatin. For example, mouse pericentric heterochromatin contains tandem DNA repeats with conserved sequence, so called major satellite repeats. From the point of view of the nucleosome positioning code approach, there would be a strong nucleosome positioning preference and the corresponding NRL equal to the length of a single repeat, 234 bp. For example, Figure 6A shows our calculation of the nucleosome occupancy and nucleosome start site probability calculated along 50 tail-to-head satellite repeats using the web server from the Segal lab [13]. The 234-bp NRL predicted from the DNA sequence would be much larger than genome-average; such a dramatic NRL difference was observed neither in our experimental nucleosome positioning dataset in mouse embryonic stem cells [28], nor in the human blood cells studied by Valouev et al [27]. What we did observe was a nucleosome start site probability broadly scattered along the satellite repeat (Figure 6B) with the periodicity of preferred nucleosome start sites equal to 10 bp (Figure 6C). The 10-bp periodicity is consistent with previous experimental observations for mouse satellite repeats performed in the pre-NGS era [80] and theoretical predictions [6]. Interestingly, the distribution of DNA fragment sizes for paired-end DNA sequencing tags mapped to satellite repeats was very broad, with many nucleosomes digested in the middle of the DNA fragment. When we have introduced a constrain that the MNase-seq DNA fragment size corresponding to a mononucleosome should be in the interval 145–150 bp, a different picture emerged (Figure 6D). Surprisingly, we have found two preferred nucleosome positions covering either nucleotides 50 through 200, or nucleotides 130 through 280 (including 40 bp from the start of the next 234-bp repeat). These two nucleosome positions apparently overlap and are mutually exclusive; the only possibility for them to realize we can think of is due to the heterogeneity of the heterochromatin, with one heterochromatin fraction having nucleosomes at positions [50–200], and another fraction having the nucleosome preferentially at [130–280]. On top of this bimodal distribution of two stable nucleosome positions which represent ∼10% of all heterochromaric nucleosomes, the majority of nucleosomes do not conform to any defined positions and only obey the 10-bp periodicity rule for the start sites. Thus, the NRL in heterochromatin is mostly independent of the DNA sequence and regulated by epigenetic variables. The finding above might be true not only for major satellite repeats, but also for other classes of repeats whose length is not a multiple of the NRL [81]. An opposite behavior was shown e.g. for AluSx repeats (313 bp), which can accommodate exactly two nucleosomes centered at well-defined positions within the repeat [25].

### Different regimes of the NRL dependence on histone concentrations

The results of this study presented above show that there are three different regimes of NRL dependence on histone concentrations and other related thermodynamic parameters. In one regime, shown in Figure 2, DNA sequence preferences are negligible (or averaged out due to comparing many unrelated DNA regions with overlapping nucleosome populations) and the major effect is due to statistical positioning of nucleosomes. This is the “classical” regime which scientists usually have in mind when speaking about statistical positioning of nucleosomes. We have systematically identified non-sequence-specific NRL dependence on the concentration of the core and linker histones, the linker protein size, the possibility of nucleosome unwrapping and the short-range and long-range internucleosome interactions (Figure 2). In this regime, increasing the nucleosome density leads to the NRL decrease due to mutual exclusions of nucleosomes. The predicted magnitude of this effect is comparable with that in the remodeler knock out experiments in *S. pombe* [31]. In addition, DNA unwrapping from the nucleosome leads to shortening of the nucleosomal DNA fragment and correspondingly shorter NRLs. Contact nucleosome-nucleosome cooperativity “glues” nucleosomes together and shortens NRL, while restrictive (anticooperative) long-range interactions make NRL larger. The latter effect becomes especially important, since only the introduction of the long-range interactions allowed our successful description of the nucleosome oscillations around yeast TSSs (Figure 3). Long-range internucleosomal interactions arise either due to the cell type-specific remodeler action or due to the intrinsic chromatin fiber structure and determine the lower NRL limit for biological species with short NRL.

In the second regime, DNA sequence preferences of the histone octamer, as well as TF-binding sequence preferences dominate over nonspecific boundary effects (Figure 5). In this regime, altering the nucleosome density or changing linker histone concentration only leads to the relative scaling of the well-defined nucleosome landscape, while the peak positions and the NRL remain mostly unaffected (Figure S4). In analogy with the mouse system studied in Figures 5 and S4, the *S. cerevisiae* chromatin reconstitution experiments of Zhang et al. [29] fall in the sequence-specific regime (Figure 2), since in these experiments NRL does not depend on histone concentration. On the other hand, the *S. pombe* remodeler knock out experiments of Hennig et al. [31] fall in the sequence-nonspecific regime, since the nucleosome density decrease is compensated by the NRL increase in these experiments (Figure 2A). Why the two types of yeast experiments belong to two different NRL variation regimes remains unclear. Perhaps, this is because *in vitro* chromatin reconstitution is more sensitive to the DNA sequence in comparison to the situation *in vivo*.

In the third regime, which we have exemplified with mouse major satellite repeats in pericentric heterochromatin, strong DNA sequence preferences are overwritten by even stronger constraints of the densely packed heterochromatin. With the same DNA sequence for all major satellite repeats, some of them harbor a nucleosome in the [50–200] position, while others in the [130–280] position. A switch between the two positions might be coupled with structural heterochromatin reorganizations. Notably, these well-defined nucleosomes account for only a ∼10% fraction of all heterochromatin nucleosomes. The rest nucleosomes are more freely arranged, just obeying the 10-bp periodicity rule. To the best of our knowledge, this is the first such report about the NRL-switch behavior in heterochromatin.

Importantly, all these regimes can be successfully described by the same biophysical model we presented here. The selection of one of the NRL regimes happens naturally at different genomic regions as a consequence of an intricate interplay of subtle changes of concentrations and geometric parameters. As a proof of principle, the NRL dependence on H1 histone concentrations has been successfully described here for different species, ranging from frog in the experiments of Oberg et al. [33] (Figure 4 A, B) to mouse strains in the experiments of Woodcock and coauthors [34] (Figure 4 D).

Finally, in all studied systems, NRL appeared to be a complicated function depending on a number of parameters, both genetic (DNA sequence) and epigenetic (changes in histone/TF/remodeler concentrations). Thermodynamic NRL regulation is thus an essential part of the control of gene expression.

## Materials and Methods

Aggregate plots for the average experimental nucleosome occupancy around TSSs, bound Pol2 and CTCF peaks were calculated as the average of nucleosome occupancies in a window of −1500 to +1500 bp around a given site using our previously published nucleosome positioning dataset with moderate MNase digestion [28]. For each gene the averaged nucleosome profile was normalized to yield the nucleosome occupancy equal to 1 at the leftmost position of the region (−1500 bp from the middle). The coordinates of CTCF binding sites and stalled Pol2 were taken as the summits of the corresponding ChIP-seq peaks determined by the Mouse ENCODE Project [74]. Theoretically predicted nucleosome patterns around TSSs, CTCF and Pol2 were calculated in the same way, using our TFnuc program [40], [53] to account for the competitive multiprotein binding and taking as input the DNA sequence preferences for nucleosomes from those determined by Segal and coauthors [13].

Heat maps of experimental nucleosome occupancy around Pol2 and CTCF binding sites were generated using a custom script in Matlab (Mathworks) by using k-means clustering or sorting according to the average nucleosome density in the region [−2000, 2000] bp. The heat map of the predicted strong nucleosome (SN) occupancy around CTCF was generated by sorting the regions according to the rightmost position of the SN.

Nucleosome distribution at major satellite repeats in mouse embryonic stem cells was determined by re-mapping our raw 50-bp paired-end reads [28] using Bowtie [82] to the genomic index containing the sequence of conserved 234 bp of major satellite repeats provided below:

“ *GGACCTGGAATATGGCGAGAAAACTGAAAATCACGGAAAATGAGAAATACACACTTTAGGACGTGAAATATGGCGAGGAAAACTGAAAAAGGTGGAAAATTTAGAAATGTCCACTGTAGGACGTGGAATATGGCAAGAAAACTGAAAATCATGGAAAATGAGAAACATCCTGACGACTTGAAAAATGACGAAATCACTAAAAAACGTGAAAAATGAGAAATGCACACTGAA* ”.

In addition, 49 first nucleotides were added to the right end of the sequence above to allow cyclic boundary conditions. Only uniquely mapped reads with up to two mismatches were retained for the analysis. Nucleosome mapping to non-repetitive regions was performed as described previously [28] using the mm9 genome assembly.

## Supporting Information

Figure S1 Average nucleosome formation energy calculated for 10,000 aligned regions containing Pol2 peaks using the software nuScore [75].
(PDF)

Figure S2 Heat map of the nucleosome density around CTCF sites bound by CTCF in ESCs based on the MNase-seq data [28]. Genomic regions were sorted by the average nucleosome density.
(PDF)

Figure S3 Trifonov's strong nucleosomes (SNs) help organize the rest of nucleosomes around CTCF binding sites, with one SN per one CTCF site.
(PDF)

Figure S4 Increasing core histone concentration does not lead to the change of the NRL at genomic regions with strong sequence preferences. Theoretically predicted nucleosome profiles in the region 3,145,000–3,147,000 of mouse chromosome 2, using as input histone octamer affinities given by the algorithm of Kaplan et al., 2009 [13]. The average nucleosome density *c*_bound_ for each of the calculations is indicated in the figure.
(PDF)

Text S1 The method of calculation of DNA-protein binding maps and the NRL.
(PDF)
