# Supplementary figures and images for: Regulation of the Nucleosome Repeat Length In Vivo by the DNA Sequence, Protein Concentrations and Long-Range Interactions
Source: PLoS Comput Biol. 2014 Jul 3;10(7):e1003698. doi: 10.1371/journal.pcbi.1003698 (PMC4081033; doi:10.1371/journal.pcbi.1003698)

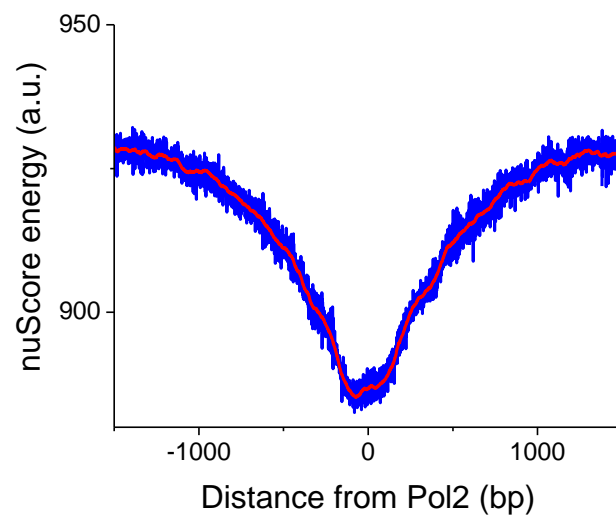

Supplement: Figure S1 — Average nucleosome formation energy calculated for 10,000 aligned regions containing Pol2 peaks using the software nuScore [75]. (PDF) [file pcbi.1003698.s001.pdf]

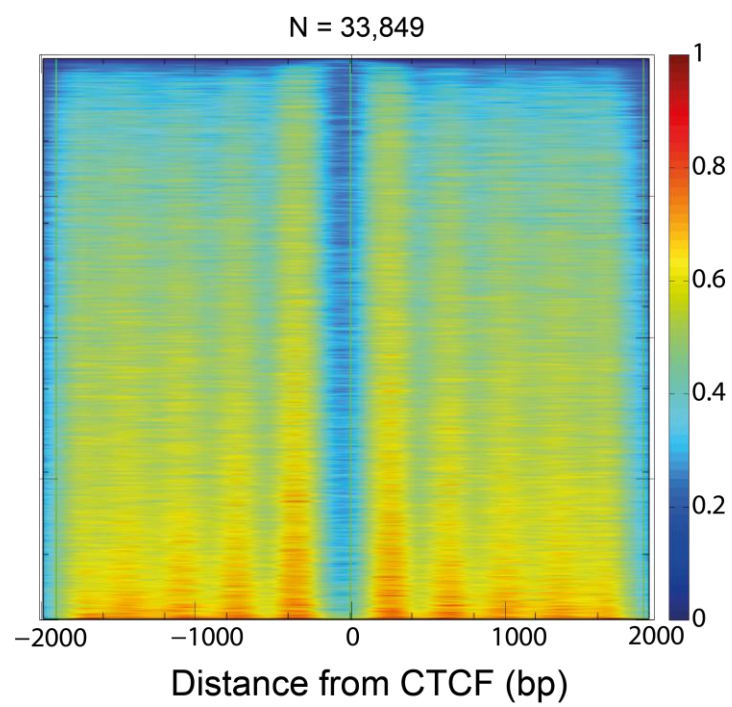

Supplement: Figure S2 — Heat map of the nucleosome density around CTCF sites bound by CTCF in ESCs based on the MNase-seq data [28]. Genomic regions were sorted by the average nucleosome density. (PDF) [file pcbi.1003698.s002.pdf]

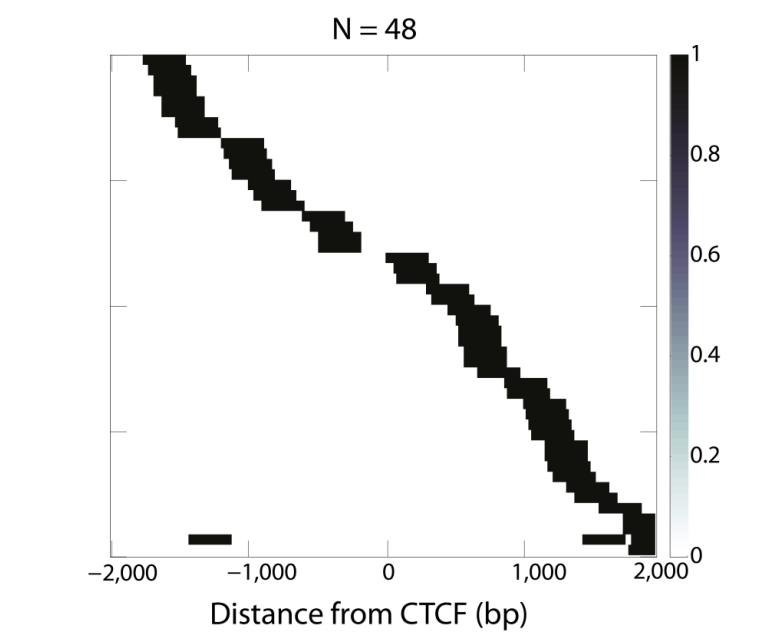

Supplement: Figure S3 — Trifonov's strong nucleosomes (SNs) help organize the rest of nucleosomes around CTCF binding sites, with one SN per one CTCF site. (PDF) [file pcbi.1003698.s003.pdf]

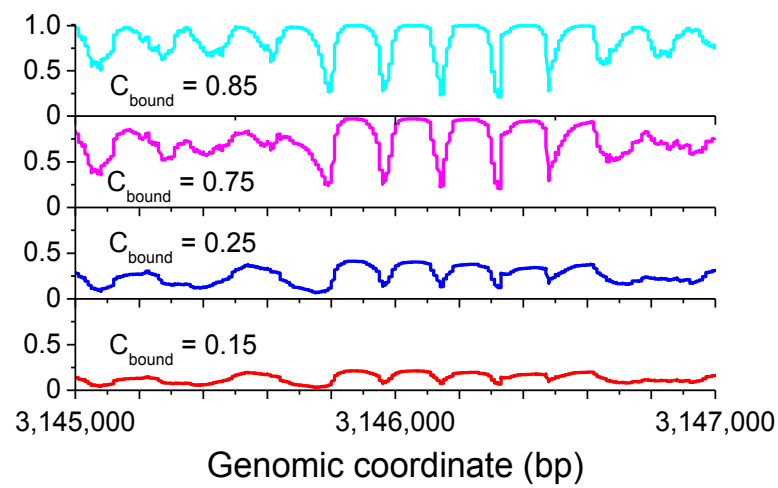

Supplement: Figure S4 — Increasing core histone concentration does not lead to the change of the NRL at genomic regions with strong sequence preferences. Theoretically predicted nucleosome profiles in the region 3,145,000–3,147,000 of mouse chromosome 2, using as input histone octamer affinities given by the algorithm of Kaplan et al., 2009 [13]. The average nucleosome density c bound for each of the calculations is indicated in the figure. (PDF) [file pcbi.1003698.s004.pdf]
