# Supplementary material for: Regulation of the Nucleosome Repeat Length In Vivo by the DNA Sequence, Protein Concentrations and Long-Range Interactions
Source: PLoS Comput Biol. 2014 Jul 3;10(7):e1003698. doi: 10.1371/journal.pcbi.1003698 (PMC4081033; doi:10.1371/journal.pcbi.1003698)
Supplement: Text S1 — The method of calculation of DNA-protein binding maps and the NRL. (PDF) [file pcbi.1003698.s005.pdf]

SUPPLEMENTARY MATERIALS

Regulation of the nucleosome repeat length *in vivo* by the DNA sequence, protein concentrations and long-range interactions

Daria A. Beshnova[1], Andrey G. Cherstvy[2], Yevhen Vainshtein[1] and Vladimir B. Teif[1,]*

[1]Deutsches Krebsforschungszentrum (DKFZ) and BioQuant, 69120 Heidelberg, Germany;

[2]Institute for Physics and Astronomy, University of Potsdam, 14476 Potsdam-Golm, Germany

*Corresponding author: Vladimir Teif (v.teif@dkfz-heidelberg.de)

The method of calculation of DNA-protein binding maps and the NRL

In this section we underline the basic steps of the mathematical procedure implemented in the main text to compute the dependence of the NRL on various thermodynamic parameters. The lattice model shown in Figure 1 can be solved mathematically either using dynamic programming or the transfer matrix formalism [1,2,3,4,5]. Here to calculate TF-DNA binding we used the dynamic programming approach, where the partition function Z for a linear DNA molecule of length N monomers can be calculated recurrently if partition functions for smaller lattices are known using the recurrent algorithm. As a result, one gets the partition function Z, which allows calculating the probability $P(n, g, h_1, h_2)$ that a protein of type g is bound starting at site n, leaving on the left and right sides correspondingly h_1 and h_2 unbound contacts (see Figure 1) with respect to its canonical binding site length $m(g)$. To account for the possibility of partial nucleosome unwrapping, the macroscopic binding constant K for the protein (or the histone octamer) whose first contact with DNA starts at position n is defined in the dependence of the number of formed bonds as a function of n, $m(g)$, g, h_1, h_2:

$$K^* = K(n, g, h_1, h_2) = \prod_{h=h_1+1}^{m(g)-h_2} k(n + h - h_1 - 1, g, h), \qquad \text{(A1)}$$

where k is the microscopic binding constant for the protein-DNA bond at position i with respect to the start of the completely bound protein binding site. Nucleosome unwrapping was calculated in the main text assuming that each base pair contributes equally to the binding constant (the so called homogeneous unwrapping potential introduced previously [4]). In order to calculate the partition function of the system let us consider the genomic region of length N binding sites, with index n numbering the first bp covered by a protein of type g, and index s numbering the last bp covered by a protein of type g ($s = n + m(g) - h_1 - h_2 - 1$). Then the partition function Z for a DNA of length s can be calculated recurrently according to Eq. A2:

$$Z_s = Z_{s-1} + \sum_{g=1}^{f}\sum_{h_1=0}^{h_{\max}}\sum_{h_2=0}^{\min[m(g)-h_1-1,h_{\max}]} c_0(g) Z_{s-m(g)+h_1+h_2-V-1} K^* +$$
$$\sum_{j=0}^{V}\sum_{g'=1}^{f}\sum_{g=1}^{f}\sum_{h_1=0}^{h_{\max}}\sum_{h_2=0}^{\min[m(g)-h_1-1,h_{\max}]}\sum_{h_1'=0}^{h_{\max}}\sum_{h_2'=0}^{\min[m(g')-h_1'-1,h_{\max}]} w(j,g',g)\cdot c_0(g)\left(Z^{+}_{s-m(g)+h_1+h_2-j}(n-m(g')+h_1'+h_2'-j,g',h_1',h_2')\right)K^* \quad \text{(A2)}$$

Here the first term is the Kornberg-Stryer term [6], while the last term account for inter-nucleosome interactions on top of the excluded volume term. Equation A2 holds with the following boundary conditions:

$$Z_s = 1 \text{ for } s < m(g) - h_1 - h_2, \quad \text{(A3)}$$

where $c_0(g)$ is the concentration of free protein of type g (c_0 is expressed in units M), $w(j,g_1,g_2)$ is the interaction potential between proteins of types g_1 and g_2 separated by j lattice units. Parameter h_{max} is the maximum possible value of nucleosome unwrapping from the left end ($h_{\max}$ is measured in DNA bp). The macroscopic binding constant $K^* = K(n, g, h_1, h_2)$ for the protein to the DNA whose first contact with DNA starts at position n, has the following boundary conditions:

$$K(n, g, h_1, h_2) = 0 \text{ for } n < 1 \text{ or } s > N. \quad \text{(A4)}$$

A given configuration with DNA positions [n, s] covered by a bound protein of type g with unbound h_1 and h_2 bp from its left and right ends respectively, is described by the following partition function:

$$Z_s^{+}(n,g,h_1,h_2) = c_0(g) Z_{s-m(g)+h_1+h_2-V-1} K^* +$$
$$\sum_{j=0}^{V}\sum_{g'=1}^{f}\sum_{h_1'=0}^{h_{\max}}\sum_{h_2'=0}^{\min[m(g')-h_1'-1,h_{\max}]}\left[Z^{+}_{s-m(g)+h_1+h_2-j}(n-m(g')+h_1'+h_2'-j,g',h_1',h_2')\right] w(j,g',g) c_0(g) K^* \quad \text{(A5)}$$

A detailed derivation of this expression as well as the history of this type of models was provided in our previous publication [1]. Equation A5 is based on the recurrent calculation of the partition function in the forward direction. Analogously, we can calculate the partial partition function backwards for the situation when the protein of type g with unwrapped h_1 and h_2 bp of the nucleosome covers region [n, s] on the DNA. This partial partition function is denoted as $Z_n^-(n,g,h_1,h_2)$. Then the product of partial partition functions $Z_s^+(n,g,h_1,h_2)\cdot Z_n^-(n,g,h_1,h_2)$ gives the sum of all states of the system where the protein of type g with unwrapped h_1 and h_2 bp covers region [n, s] on the DNA. This expression has to be divided by $c_o(g)\cdot K(n,g,h_1,h_2)$ because the forward and reverse partition functions take into account our protein of interest twice [1]. Finally, in order to find the probability of a TF binding event to the DNA we have to divide this expression by the total partition function Z_n of the system. Then the probability P that the protein of type g with unwrapped h_1 and h_2 bp starts at position n along the DNA is given by the following expression:

$$P(n,g,h_1,h_2)=\frac{Z_s^+(n,g,h_1,h_2)\cdot Z_n^-(n,g,h_1,h_2)}{Z_N\cdot c_o(g)\cdot K(n,g,h_1,h_2)}. \quad \text{(A6)}$$

The probability that a specific DNA base pair is occupied by the protein of type g is given as follows:

$$C(n,g)=\sum_{g=1}^{f}\sum_{h_1=0}^{m(g)-1}\sum_{h_2=0}^{m(g)-h_1-1}\sum_{i=n-m(g)+h_1+h_2+1}^{n}P(i,g,h_1,h_2). \quad \text{(A7)}$$

The total concentration of the protein of type g is a sum of its free and bound concentrations:

$$Ctotal(g)=c_0(g)+\sum_{n=1}^{N}C(n,g)\cdot\frac{C_N}{N\cdot m(g)}, \quad \text{(A8)}$$

where C_N is the concentration of DNA of length *N* bp.

Equation A8 was used for the calculations of [Linker]/[NCP] relation on Figures 3-4. The nucleosome occupancy profiles were calculated with the help of the *TFnuc* software (will be made available online at http://generegulation.info). The NRL was determined from a linear fit of the detected peak positions versus the nucleosome number (see Figure 1D and E of the main text). Up to 4 peaks could be identified in this analysis.

Supplementary references

1. Teif VB, Rippe K (2012) Calculating transcription factor binding maps for chromatin. Brief Bioinform 13: 187-201.
2. Teif VB, Erdel F, Beshnova DA, Vainshtein Y, Mallm JP, et al. (2013) Taking into account nucleosomes for predicting gene expression. Methods 62: 26-38.
3. Teif VB, Rippe K (2011) Nucleosome mediated crosstalk between transcription factors at eukaryotic enhancers. Phys Biol 8: 04400.
4. Teif VB, Ettig R, Rippe K (2010) A lattice model for transcription factor access to nucleosomal DNA. Biophys J 99: 2597-2607.
5. Teif VB, Beshnova DA, Vainshtein Y, Marth C, Mallm JP, et al. (2014) Nucleosome repositioning links DNA (de)methylation and differential CTCF binding during stem cell development. Genome Research: Published online May 8, 2014. DOI: 2010.1101/gr.164418.164113.
6. Kornberg RD, Stryer L (1988) Statistical distributions of nucleosomes: nonrandom locations by a stochastic mechanism. Nucleic Acids Res 16: 6677–6690.
